# Psychosocial and clinical impact of COVID-19 pandemic and its relationship to the quality of life in patients with rheumatoid arthritis: a cross-sectional study, Egypt

**DOI:** 10.1186/s43045-022-00184-2

**Published:** 2022-02-23

**Authors:** Mervat S. Hassan, Dalia I. Mostafa, Enas I. Abdelhady, Shymaa A. Sarhan, Mohamed Abdelghani, Dina A. Seleem

**Affiliations:** 1 Psychiatry Department, Faculty of Medicine, Zagazig University, Zagazig, Egypt; 2 Rheumatology and Rehabilitation Department, Faculty of Medicine, Zagazig University, Zagazig, Egypt; 3 2015-2016 Hubert H. Humphrey Fellowship, Department of Psychology, Virginia Commonwealth University, Richmond, USA

**Keywords:** Rheumatoid arthritis, Quality of life, COVID-19 pandemic, Egypt

## Abstract

**Background:**

Data have been pouring on the impact of the COVID-19 pandemic on patients with chronic diseases. This study aimed to address the relationship between the perceived fears of COVID-19 virus (FCV), psychological status, and quality of life (QoL) among patients with rheumatoid arthritis (RA) during the pandemic. This study included 100 patients with RA and an equal number of control subjects, who were recruited from Zagazig University rheumatology outpatient clinics, Egypt. All subjects were interviewed using the fear of COVID-19 scale (FCV-19S), Symptom Checklist-90 scale (SCL-90), and World Health Organization Quality of Life Scale (WHOQOL-BREF). Patients were additionally assessed using the Disease Activity Score 28 (DAS28) and Modified Health Assessment Questionnaire (MHAQ).

**Results:**

There were significant differences between both groups in all QoL domains and most psychological dimensions. Most patients with RA experienced moderate-to-high disease activity and mild-to-moderate functional impairment (85% and 80%, respectively). FCV was correlated with the number of family members (*P*-value 0.020), and obsessive-compulsive (*P*-value 0.006), interpersonal sensitivity (*P*-value 0.035), hostility (*P*-value 0.017), phobia (*P*-value 0.010), and psychoticism (*P*-value 0.034) symptoms. Moderate-to-high disease activity was associated with reduced psychological QoL. Patients with moderate-to-severe functional impairment had worse QoL in all domains (except social). Prolonged illness duration was associated with worse social QoL.

**Conclusions:**

QoL was adversely affected in patients with RA during the pandemic. There was a robust relationship between FCV and the emergence of psychological symptoms. RA-related clinical factors like illness duration, disease activity, and functional disability were associated with reduced QoL in those patients.

## Background

Since the initial outbreak of SARS-CoV-2 from Wuhan Province in China in December 2019 [1], the epidemic had a rapid global spread worldwide which led the World Health Organization (WHO) to declare the disease a global health emergency at the end of January 2020 and a pandemic in March 2020 [2]. During the outbreak, a wide array of psychosocial symptoms was observed in various populations, such as the fear of contracting an infection or the fear of death as well as feelings of despair and social isolation [3–5].

Given the lack of specific protocols for the treatment of COVID-19 infection, the current therapies were primarily supportive, even though several agents are now waiting for approval for the treatment of this life-threatening disease. RA is a chronic autoimmune disease with a progressive and debilitating course, and common psychological comorbidities [6]. The COVID-19 pandemic is expected to change the treatment strategies of a chronic disorder like RA, whose patients are at an increased infectious risk compared to the general population owing to their immune suppression of being an autoimmune disease as well as the effect of medications like corticosteroids and other immunosuppressive drugs [7].

Being at higher risk to get the COVID-19 infection, the fear of COVID-19 (FCV) among patients with RA was supposed to be intensified. It was well reported that FCV had a negative impact on overall well-being, with intensified levels of depression, anxiety, and stress [8]. Likewise, psychiatric comorbidities (e.g., depression) would be associated with higher disease activity, including reduced pain tolerance [9], and increased levels of functional disability, which eventually resulted in poorer long-term outcomes, and reduced quality of life (QoL) [10]. The European League Against Rheumatism (EULAR) had reported increased levels of anxiety in patients receiving steroids and other RA medications including methotrexate and hydroxychloroquine during the current pandemic [11].

Although it was hypothesized that the COVID-19 pandemic and its associated correlates would adversely influence the physical and psychological status of patients with chronic diseases [12], research investigating the relationship between the perceived FCV and other associated psychological symptoms secondary to the pandemic, and the QoL in patients with RA in Egypt, is still lagging. The aim of this study was primarily to assess the psychosocial and clinical impact of the COVID-19 pandemic on patients with RA in Egypt and to compare these findings with their healthy counterparts.

## Methods

### Study setting, design, and sampling

This case-control study was conducted at the rheumatology outpatient clinics of the Zagazig University hospitals in Sharkia province, Egypt, from October 1st, 2020, to March 31st, 2021. The sample size was calculated according to a 95% confidence interval (CI), at 80% power of the study, the ratio of sample size 1:1. The patient group included a total of 100 subjects diagnosed as patients with RA if they had scored 6 or more points based on the rheumatoid arthritis criteria of the American College of Rheumatology (ACR) and the European League Against Rheumatism (EULAR) collaborative initiative [13]. Patients aged from 18 to 60 years, of both sexes, and able to give consent, were included in the study. An equal number of healthy subjects, who were age and sex-matched, were enrolled as a control group. All subjects were consecutively selected using a convenience sampling method. All participants with a confirmed history of psychiatric disorders (before the pandemic), based on the history taken or their medical records, were excluded from this study. Moreover, participants with concurrent major neurocognitive disorders, intellectual disabilities, and/or substance use disorders were excluded, as their presence would affect the credibility to participate in the study. All procedures were conducted within the ethical guidelines outlined in the declaration of Helsinki and its later amendments, and all participants were requested to sign an informed consent after explaining the study objectives and procedures.

### Assessment measures

Using a semi-structured checklist, all study participants were interviewed to collect demographic and COVID-19-related data. The COVID-19-related data included questions about whether the participants or their close family members were previously infected with the COVID-19 virus, history of COVID-19 mortality among close relatives, and whether they were compliant or not with the COVID-19 precautions like social distancing or protective equipment measures.

#### Psychological assessment of study participants

##### Symptom Checklist-90 scale (SCL-90)

SCL-90 was used as a descriptive measure of psychopathology detecting the current symptom severity in various patient populations. The concurrent psychological symptoms in patients with RA during the pandemic, screened by the SCL-90 checklist, might be considered as potential confounders related to the reaction to the pandemic or the illness itself. SCL-90 included ninety questions answered by the participants on a five-point Likert scale ranging from 0 (none) to 4 (extreme) representing the nine symptom dimensions for the past 1 month: namely, somatization, obsessive-compulsive, interpersonal sensitivity, depression, anxiety, hostility, phobic anxiety, paranoid ideation, and psychoticism. Additionally, there were three global indices: namely Global Severity Index (GSI), which was the mean sum of the 90 items, Positive Symptom Distress Index (PSDI), which was the mean sum of only the above-zero items, and lastly, Positive Symptoms Total (PST), which was the number of the above-zero items [14, 15].

##### Fear of COVID-19 scale (FCV-19S)

FCV-19S was a new short valid seven-item psychometric scale used to assess the participants’ intensified anxiety and fear of COVID-19 infection. The participants used a five-item Likert-type response to indicate their level of agreement with each statement. The score for each question ranged from 1 (strongly disagree) to 5 (strongly agree), with a total score ranging from 7 to 35. The higher the score, the greater the fear of coronavirus-19 [16, 17].

##### World Health Organization quality of life scale (WHOQOL-BREF)

WHOQOL-BREF was used to assess the quality of life (QoL). It was composed of a total of 26 questions based on a four-domain structure: physical health, psychological, social relationships, environment. Items were answered on a 5-point scale with a 2-week timeframe. The mean of items within each domain was multiplied by four to yield the domain score (ranging from 0 to100), and the higher scores indicate higher QoL [18, 19].

#### Clinical assessment of patients with RA

The following measurement tools were applied to assess the clinical impact of the pandemic as well as the COVID-19-related factors like associated fear, anxiety, and depression on the physical status, disease activity, and functioning of patients with rheumatoid arthritis (RA). In this study, the clinical impact was assessed using the following measures:

##### Disease activity score 28 (DAS28)

DAS28 was used to assess RA disease activity [20]. It is a short scale derived from the original DAS with the inclusion of fewer joints. It consisted of a 28 tender joint count (ranged from 0 to 28), a 28-swollen joint count (ranged from 0 to 28), ESR, and GH on a VAS scale (ranged from 0 to 100). DAS28 was scored as a continuous index ranging from 2 to 10, where the patients were divided into four disease activity grades (remission, low, moderate, and high activity) [21].

#### Modified health assessment questionnaire (MHAQ)

MHAQ was a self-reported outcome questionnaire and used to measure the functional status of patients with RA. It included questions about the perceived patient’s satisfaction regarding the same daily activities, as well as the change in the degree of difficulty. Patients were divided into normal, mild, moderate, and severe according to their functional status [22].

### Statistical analysis

SPSS program (Statistical Package for Social Science) version 18.0 was used to analyze the collected data. The differences between the qualitative variables between groups were estimated using the Chi-square test (*χ*^*2*^). Paired Student test (*t-*test) was used for comparison between two groups for continuous variables and the Mann-Whitney-U (MWU) test for non-normally distributed data. To evaluate the correlation between two variables that had a linear relationship, Pearson’s correlation coefficient was applied. Results were considered significant when their probability was less than 5% (*P*<0.05).

## Results

### Demographic and psychological characteristics of study participants

The study included a total of two-hundred subjects (one hundred patients with RA and an equal number of normal subjects as a control group). The mean age of RA patients was 43.4 ± 10.0 years while that of the control group was 41.4 ± 10.2 years. About one-fourth of patients with RA reported that they or one of their close relatives were previously confirmed as COVID-19 patients. There were significant differences between patients with RA and the control group in terms of education, working status, comorbid organic diseases, and compliance with COVID-19 precautions. Patients with RA, compared with their normal counterparts, were more likely to be less educated ((53% vs 11%), unemployed (87% vs 47%), having a history of comorbid organic diseases (32% vs 19%), and more compliant with COVID-19 precautions (75% vs 33%).

The means of physical, psychological, social, and environmental QoL domains of patients with RA were significantly lower than those reported by control subjects. Similarly, the mean scores of symptoms of somatization, obsession and compulsion, depression, anxiety, phobia, paranoia, and psychoticism were much higher among patients with RA than control subjects. However, despite higher in patients with RA, there was no difference between both groups in terms of FCV, as illustrated in Tables 1 and 2.

**Table 1:** Demographic and COVID-19 related factors of patients with RA and Control Subjects

Variable	Control	Cases	Total	***Х***^**2**^/t-test	***P***-value
	*N* (%), Mean (SD)				
Age	41.4 (10.2)	43.4 (10.0)	43.7 (10.5)	-4.20	0.374
Gender					
Female	91 (91)	92 (92)	183 (91.5)	0.06	0.800
Male	9 (9)	8 (8)	17 (8.5)		
Education					
Non-to-low	11 (11)	53 (53)	64 (32)	40.53	**<0.001**
Higher	89 (89)	47 (47)	136 (68)		
Working status					
Employed	53 (53)	13 (13)	66 (33)	36.18	**<0.001**
Unemployed	47 (47)	87 (87)	134 (67)		
History of medical comorbidities					
No	81 (81)	68 (68)	149 (74.5)	4.45	**0.035**
Yes	19 (19)	32 (32)	51 (25.5)		
Number of family members	2.5 (0.6)	2.8 (0.9)	2.6 (0.8)	-1.400	0.163
Self or close relative history of COVID-19 affection					
No	78 (78)	75 (75)	153 (76.5)	5.604	0.118
Yes	22 (22)	25 (25)	47 (23.5)		
Compliance with COVID-19 precautions					
No	67 (67)	25 (25)	92 (46)	35.51	**<0.001**
Yes	33 (33)	75 (75)	108 (54)		
FCV-19S	17.3 (5.0)	18.9 (8.4)	18.1 (6.9)	-1.61	0.110

Significant values at *P* < 0.05 are presented as bold

*QoL* quality of life, *SCL-90* Symptom Checklist-90 scale, *OC* obsessive-compulsive, *GSI* Global Severity Index, *PST* positive symptoms total, *PSDI* Positive Symptom Distress Index

**Table 2:** Associated psychological symptoms and quality of life in patients with RA and Control Subjects

Variable	Control	Case	Total	***t***-test	***P***-value
	Mean (SD)				
QOL (Physical)	63.6 (16.3)	43.23 (14.4)	53.5 (18.4)	9.38	**<0.001**
QOL (Psychological)	57.3 (16.2)	50.8 (12.9)	54.0 (15.0)	3.17	**0.002**
QOL (Social)	78.7 (32.0)	54.6 (18.2)	66.6 (28.6)	6.54	**<0.001**
QOL (Environmental)	51.3 (15.5)	45.5 (14.2)	48.4 (15.1)	2.80	**0.006**
QOL (Total)	59.8 (14.0)	47.3 (11.8)	53.6 (14.4)	6.86	**<0.001**
	Mean (SD) Range			**MWU**	
SCL (Interpersonal Sensitivity)	12.1 (6.5) 2-30	13.4 (8.2) 1-36	12.8 (7.4) 1-36	-0.93	0.352
SCL (Depression)	17.3 (8.7) 4-47	20.5 (10.7) 2-51	18.9 (9.9) 2-51	-2.15	**0.032**
SCL (Anxiety)	12.0 (6.9) 2-27	14.5 (8.3) 2-39	13.3 (7.7) 2-39	-2.06	**0.039**
SCL (Hostility)	6.3 (3.9) 0-18	7.4 (5.7) 0-24	6.9 (4.9) 0-24	-0.55	0.582
SCL (Phobic)	5.9 (4.8) 0-19	9.0 (6.6) 0-28	7.4 (6.0) 0-28	-3.42	**0.001**
SCL (Paranoid)	6.0 (4.1) 0-20	8.4 (5.7) 1-24	7.2 (5.1) 0-24	-3.89	**0.004**
SCL (Psychotic)	6.9 (6.2) 0-29	11.1 (9.4) 2-40	9.0 (8.2) 0-40	-2.09	**0.002**
	Mean (SD)			***t*-test**	
SCL (Somatization)	16.7 (8.3)	23.7 (8.7)	20.2 (9.2)	-5.78	**<0.001**
SCL (OC)	14.3 (5.8)	18.3 (7.7)	16.3 (7.1)	-4.17	**<0.001**
Total	107.8 (51.6)	138.0 (67.5)	122.9 (60.5)	-3.45	**0.001**
GSI	1.2 (0.6)	1.5 (0.7)	1.4 (0.7)	-3.45	**0.001**
PST	56.3 (18.6)	66.6 (19.9)	61.5 (19.9)	-3.78	**<0.001**
PSD	1.8 (0.4)	2.0 (0.6)	1.9 (0.5)	-1.79	0.075

Significant values at *P* < 0.05 are presented as bold

*QoL* Quality of life, *SCL-90* Symptom Checklist-90 scale, *OC* Obsessive-compulsive, *GSI* Global Severity Index, *PST* Positive Symptoms Total, *PSDI* Positive Symptom Distress Index, *QoL* Quality of life

### Perceived fear of COVID-19 infection, demographic, clinical, and psychological factors in patients with RA

**Fig. 1:**
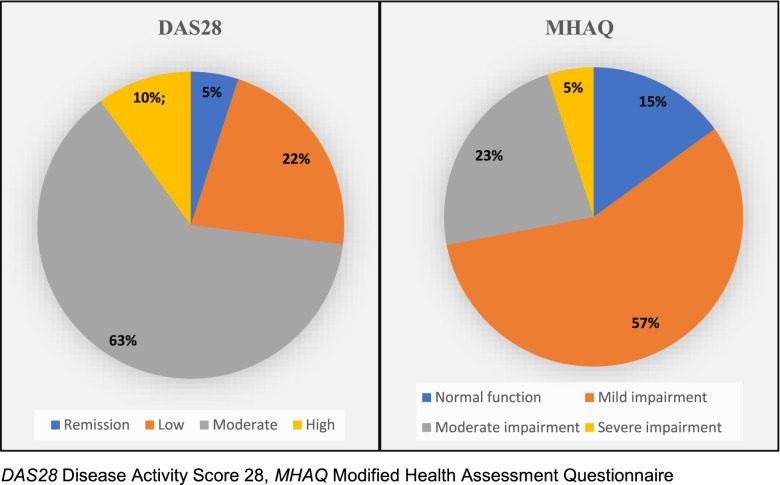
Clinical characteristics of patients with RA

It was found that about 80%, 86%, and 5% of the patients with RA received steroids, disease-modifying antirheumatic drugs (DMARDs) as hydroxychloroquine or methotrexate, and immunosuppressants as cyclosporine, respectively. Moreover, 17% of patients stated that they were not compliant with their medications during the previous 6 months before the study. As shown in Fig. 1, most patients with RA had experienced moderate-to-high disease activity (85%), as scored by DAS28, and mild-to-moderate functional impairment (80%), according to MHAQ. Moreover, the perceived FCV was correlated with increased number of family members (*r* 0.23, *P*-value 0.020), and symptoms of obsession and compulsion (*r* 0.27, *P*-value 0.006), interpersonal sensitivity (*r* 0.21, *P*-value 0.035), hostility (*r* 0.24, *P*-value 0.017), phobia (*r* 0.26, *P*-value 0.010), and psychoticism (*r* 0.20, *P*-value 0.034), as illustrated in Tables 3 and 4.

**Table 3:** Correlation between perceived fear of COVID-19 infection, and demographic, clinical, and COVID-19-related factors of patients with RA

FCV-19S Age	Pearson correlation (***r***)		***P***-value
	−0.06		0.574
Number of family members	0.23		**0.020**
	*M* (SD)	**MWU**	
Gender		−1.16	0.248
Female	18.6 (8.4)		
Male	22.1 (6.9)		
Marital status		−0.82	0.410
Married	19.1 (8.4)		
Not married	17.2 (8.3)		
Education		−1.40	0.161
Non-to-low	17.8 (8.8)		
Higher	20.2 (7.7)		
Working status		−0.58	0.564
Employed	19.8 (7.8)		
Unemployed	18.8 (8.5)		
History of medical comorbidities		−0.18	0.856
No	18.9 (8.0)		
Yes	19.0 (8.4)		
Compliance with COVID-19 precautions		−0.69	0.492
No	17.8 (8.2)		
Yes	19.3 (8.4)		
Current DAS28		−0.29	0.770
Remission-to-low	19.12 (7.2)		
Moderate to-high	18.8 (8.7)		
MHAQ		−0.75	0.452
Normal-to-mild	19.4 (8.4)		
Moderate-to-severe	17.7 (8.3)		

Significant values at *P* < 0.05 are presented as bold

*FCV-19S* fear of COVID-19 scale, *DAS28* Disease Activity Scale 28, *MHAQ* Modified Health Assessment Questionnaire, *MWU* Mann-Whitney *U* test

**Table 4:** Correlation between perceived fear of COVID-19 infection and associated psychological symptoms and quality of life in patients with RA

Variable	FCV	***P***-value
	Pearson correlation (***r***)	
SCL (somatization)	0.12	0.224
SCL (OC)	0.27	**0.006**
SCL (interpersonal sensitivity)	0.21	**0.035**
SCL (depression)	0.17	0.094
SCL (anxiety)	0.18	0.077
SCL (hostility)	0.24	**0.017**
SCL (phobic)	0.26	**0.010**
SCL (paranoid)	0.20	0.051
SCL (psychoticism)	0.20	**0.043**
Total	0.22	**0.028**
GSI	0.22	**0.028**
PST	0.21	**0.038**
PSDI	0.18	0.071
QOL (physical)	−0.01	0.953
QOL (psychological)	0.06	0.545
QOL (social)	0.13	0.202
QOL (environment)	0.01	0.957
QOL (total)	0.04	0.680

Significant values at *P* < 0.05 are presented as bold

*FCV-19S* Fear of COVID-19 scale, *SCL-90* Symptom Checklist-90 scale, *OC* Obsessive-compulsive, *GSI* Global Severity Index, *PST* Positive Symptoms Total, *PSDI* Positive Symptom Distress Index, *QoL* quality of life

QoL was found to be likely affected by the RA-related clinical factors. Moderate-to-high disease activity was associated with lesser scores of psychological and total QoL. Also, patients with moderate-to-severe functional impairment reported worse QoL in all domains (except social). Lastly, increased illness duration was associated with worse social QoL (*r* 0.26, *P*-value 0.010), as illustrated in Table 5.

**Table 5:** Relationship between illness related clinical factors and quality of life in patients with RA

	QoL (Physical)	QoL (Psychological)	QoL (Social)	QoL (Environment)	QoL (Total)
	Mean (SD)				
Current DAS28					
Remission-to-low	47.1 (15.4)	56.3 (11.2)	56.2 (18.5)	50.3 (14.2)	51.6 (11.0)
Moderate to-high	42.1 (13.9)	49.1 (13.0)	54.1 (18.2)	44.0 (14.0)	46.0 (11.7)
*t*-test, *P*-value	1.45, 0.149	2.43, **0.017**	0.47, 0.639	1.87, 0.064	2.03, **0.045**
MHAQ					
Normal-to-mild	46.8 (13.4)	52.7 (12.7)	56.3 (18.6)	47.7 (14.5)	49.8 (11.4)
Moderate-to-severe	34.1 (12.7)	45.7 (12.3)	50.3 (16.7)	39.7 (11.7)	40.9 (10.2)
*t*-test, *P*-value	4.34, **<0.001**	2.51, **0.014**	1.48, 0.143	2.59, **0.011**	3.59, **0.001**
	**Pearson correlation (*r*),** ***P*-value**				
Duration of illness	−0.02, 0.810	−0.01, 0.991	0.26, **0.010**	0.09, 0.366	0.08, 0.444

Significant values at *P* < 0.05 are presented as bold

*QoL* quality of life, *DAS28* Disease Activity Score 28, *MHAQ* Modified Health Assessment Questionnaire

## Discussion

This study aimed primarily to assess the impact of the COVID-19 pandemic on patients with RA. Those patients showed significantly higher adherence to COVID-19 regulations than the healthy ones. Being chronic patients with a higher risk of poor COVID-19 outcomes, they were supposed to follow strict social distancing measures (24). However, this strict social distancing would crucially increase loneliness, exacerbate anxiety, and depression among those patients.

The pandemic had led to increased rates of fear, anxiety, depression, and social isolation in the various populations, which all would be magnified in patients with chronic diseases [12, 23]. It was observed that during the initial phase of the coronavirus outbreak, in the general population, increased numbers of individuals reported higher levels of anxiety and depressive symptoms [24]. Another study of patients with RA in New York City reported increased fatigue, stress, anxiety, cognitive worsening, and musculoskeletal symptoms. In addition, patients expressed worries about medication changes, family, work, and finances [25]. In the current study, patients with RA had more psychological symptoms than control subjects. Likewise, FCV was correlated with most psychological symptom dimensions of SCL-90, as well as the number of family members. This would be, in part, attributed to the financial burden related to the economic influence of the pandemic on the large-sized families, as well as the propensity of contracting COVID-19 infection that would eventually add additional psychological stress on the individuals with RA during the pandemic [26]. Moreover, it was intuitively supposed that even before the COVID era, depression, and anxiety symptoms were frequently observed in patients with RA [27]. Nonetheless, the increased severity of psychological symptoms during the pandemic would be explained in the view of the early reports since the pandemic, which suggested that those patients are at a higher risk of respiratory failure and death from COVID-19 [28, 29]. The patients were warned by the Centre for Disease Control (CDC) to be more cautious during the pandemic owing to their reduced immune status [30]. Also, patients with RA would face unique challenges during the pandemic rather than the concerns of contracting the infection itself. Early propaganda on hydroxychloroquine, one of the drugs used to treat RA, promoted as a potential COVID-19 prophylactic and treatment agent, resulted in drug shortages for patients with rheumatic diseases, including RA. Other difficulties included problems accessing care due to the lockdown [23, 31].

In this study, patients with RA, during the pandemic, experienced a reduced quality of life for RA when compared to healthy individuals. RA was a chronic disabling condition that could impair work productivity and activities of daily living and compromise the well-being of patients [32]. It was found that patients with RA would experience a certain degree of functional impairment (in this study, 80% of patients had mild-to-moderate functional impairment), and this impairment would increase health care expenses, decrease the quality of life, and strongly predict depression and increased suicide risk [33].

RA itself had a substantial effect on QoL. This study showed a strong relationship between the illness duration, activity and functional status, and QoL. In RA, it was postulated that the burden of the disease would depend on both emotional and physical components with subsequent impact on overall well-being [34]. In line with these results, a study conducted on 170 patients with RA found that patients with worse disease activity were likely to experience more severe depressive symptoms [35]. Therefore, routine assessment of the disease’s overall impact on a patient’s life, including assessment of emotional status and QoL, was recommended in several recent guidelines. These guidelines advocated that evaluation of treatment success should not only be seen from a clinical perspective but should include a patient’s overall quality of life. Thus, a shift should be done from merely the strict medical/symptom-related model to restoring or at least improving the quality of life [36].

### Limitations

This study had a few limitations. The cross-sectional design of this study and the potentially brief duration of assessment would infer the conclusion of the causality of the impact of COVID-19 on those populations. However, it would be argued that this study would be one of few studies, if any, in Egypt which addressed QoL and other related factors among patients with RA during the COVID-19 pandemic. Future large-sized longitudinal studies, perhaps including psychological interventions, would be warranted. Another concern was the lack of multicentric character that would limit the generalizability of results. The authors advocated that the Zagazig University Hospitals, located in Sharkia Province, were one of the largest general health facilities serving around seven million inhabitants in the eastern delta, Egypt.

## Conclusions

Psychological status and QoL were adversely affected in patients with RA during the pandemic. RA-related clinical factors like illness duration, disease activity, and impaired functional status were all associated with reduced QoL. Moreover, there was a robust relationship between the perceived fear of COVID-19 and the emergence of more psychological symptoms in patients with RA. The study results would shed the light on the potential impact of the pandemic on the QoL of patients with chronic diseases, particularly RA. Also, it highlights the need to develop routine screening interviews for the associated psychological and physical health-related issues that should be regularly applied during and after this pandemic. It should include early monitoring, assessment, and management for the potentially emerging psychological symptoms.

## Data Availability

The data collected and analyzed in this study are available from the corresponding author upon reasonable request.

## Abbreviations

FCV: Fear of COVID-19 virus
FCV-19S: Fear of COVID-19 scale
GSI: Global Severity Index
PSDI: Positive Symptom Distress Index
QoL: Quality of life
RA: Rheumatoid arthritis
SCL-90: Symptom Checklist-90 scale
WHOQOL-BREF: World Health Organization Quality of Life Scale
